# Verrucous Carcinoma of Buccal Mucosa in Female: A Rare Case Report of Traumatic Tooth Origin

**DOI:** 10.1155/2021/6673038

**Published:** 2021-06-11

**Authors:** V. Keerthi Narayan

**Affiliations:** Department of Oral Pathology and Microbiology, Thai Moogambigai Dental College and Hospital, Dr. MGR Educational and Research Institute, 600107, Chennai, Tamil Nadu, India

## Abstract

Verrucous carcinoma or Ackermann's tumor is considered a low-grade variant of squamous cell carcinoma frequently presenting at the oral mucosa and skin. Oral verrucous carcinoma clinically presents as a proliferative or cauliflower-like lesion or as ulceroproliferative lesion on the buccal mucosa followed by other sites such as the gingiva, tongue, and hard palate. Tobacco in both smoking and smokeless form, alcohol, and opportunist viral infections are the most associated etiologies in most of the reported literature cases. Here, in this paper, we discuss a rare case scenario of a 52-year-old female diagnosed with verrucous carcinoma of the left buccal mucosa with constant traumatic irritation caused by tooth as etiology for the occurrence of lesion, though verrucous carcinoma is described as a benign lesion with minimum aggressive potential but long-standing cases have shown transformation into squamous cell carcinoma. Therefore, early diagnosis and surgical excision of the lesion are the most appropriate treatment modality of verrucous carcinoma.

## 1. Introduction

Cancer of the head and neck region is of significant public health importance in India. Most often, it is diagnosed at advanced or late stages resulting in extremely low and poor treatment outcomes with considerable financial constrain [1]. Oral cancer often manifests itself in about 45%-48% of cases as ulcers or ulcerated tumors. Owing to its wide range of causative factors and unpredictable presenting features, the diagnosis of oral ulcerative lesions might be quite challenging. In initial cases, these carcinomatous ulcers can be confused with other pathological entities and often misdiagnosed as nonneoplastic ulcerative lesion. Such lesions lasting for two weeks or longer often create a dilemma due to its chronicity and unresponsive behaviour to various treatments prescribed [2].

In order to arrive at a definitive diagnosis, earlier detection of oral cancer followed by laboratory tests and additional investigations to narrow the diagnosis offers the best chance for long term survival and has the potential to improve treatment outcomes and make healthcare services affordable [3–5]. In this paper, we discussed an unusual presentation of verrucous carcinoma (VC) of the buccal mucosa in a female patient with rapid progression of the disease even after the removal of etiological agent suggesting a new variation in a disease process. In addition to this unfamiliar etiological factor and course of the disease, these findings could shed new light on the possible pathogenesis of verrucous carcinoma.

## 2. Case Report

### 2.1. About the Case

A 52-year-old female patient visited the Outpatient Department of Our Dental College and Hospital with a chief complaint of a nonhealing ulcerated tissue on the left lower back tooth region for the past 6 months, associated with pain and burning sensation during intake of spicy or hot food items occasionally. She developed continuous severe burning sensation accompanied by pain since 1 week in the same region. No relevant past medical, dental, occupational, and habit history was observed in relation with the chief complaint.

### 2.2. On Intraoral Examination

#### 2.2.1. Visit 1

**Figure 1:**
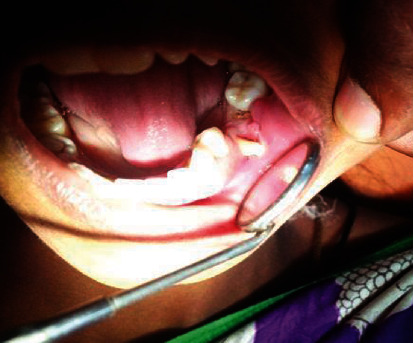
Clinical photograph showing a solitary, firm, ulcerative tissue measuring 2 × 2 centimetres on the alveolar mucosa near the premolar region.

**Figure 2:**
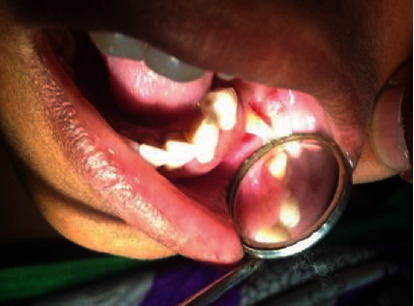
Clinical photograph showing the stretching of the cheek mucosa a tooth root found extruding from the socket impinging on the soft tissue counterpart surrounding areas.

**Figure 3:**
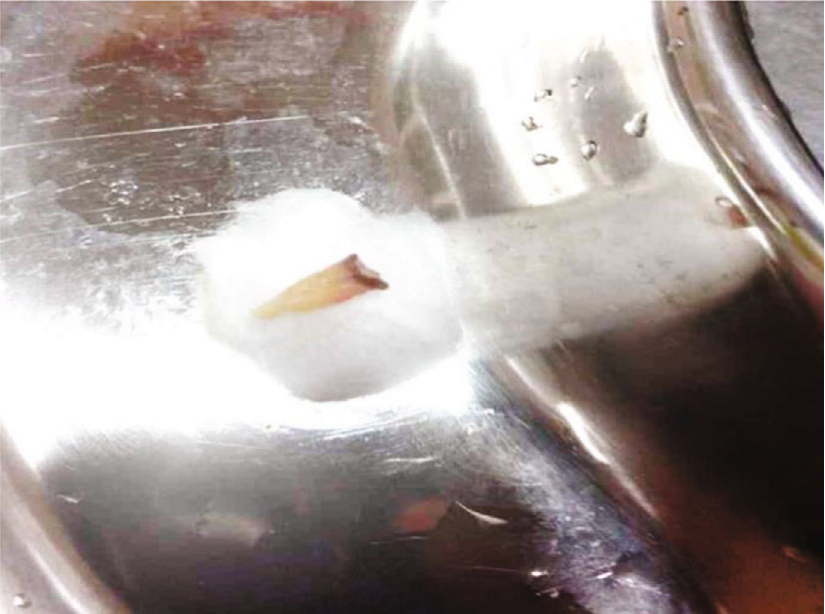
Clinical photograph showing a completely removed tooth under local anaesthesia.

On soft tissue examination, a solitary, firm, ulcerative tissue measuring 2 × 2 centimetres was found on the alveolar mucosa near premolar region separated from it by clinically healthy mucosa. On stretching the cheek mucosa, a tooth root was found extruding from the socket impinging on the soft tissue counterpart surrounding areas (Figures 1 and 2). No other lesions were observed on either side of the oral mucosa. After comprehensive intraoral examination followed by analysis of the past dental and relevant history, an interim diagnosis of traumatic ulcer was given in association with the tooth as the source of origin. Patient consent was obtained before proceeding with the treatment. Extraction of the tooth root was performed under local anaesthesia (Figure 3). The procedure was uneventful, and postoperative instructions were given for the same. The patient was normal and freed of complaints during postoperative follow-up made after 15days.

#### 2.2.2. Visit 2

**Figure 4:**
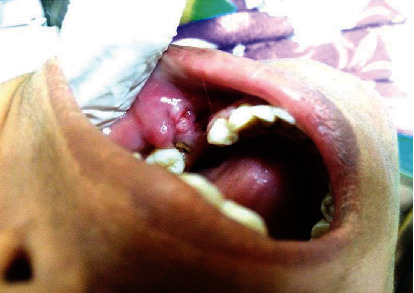
Clinical photograph showing nodular proliferous ulcerative growth measuring 3 × 2 centimetres on the alveolar mucosa.

The patient reported again to the outpatient department with similar complaints of pain and severe burning sensation after 3 months in the same region. On intraoral examination, nodular proliferous ulcerative growth measuring 3 × 2 centimetres was found on the alveolar mucosa near the premolar-molar region with focal areas of white spots (Figure 4). She was referred immediately to our oral pathology department followed by prescription of topical application of antiviral drugs (2% acyclovir) for a week. As the lesion did not show signs of regression, routine blood investigations were carried out to rule out complications following biopsy and were found to be normal. An incisional biopsy was advised for the same with differential diagnosis of focal epithelial hyperplasia; traumatic fibroma, verruciform leukoplakia, verruca vulgaris, verrucous carcinoma, and ulcerative squamous cell carcinoma were suggested.

### 2.3. Histopathological Analysis

Histopathological analysis showed a broad bulbous rete pegs with pushing margins in many areas along with features of mild dysplasia and few areas of parakeratin plugging. There were also few areas of severe dysplasia without significant break in the basement membrane and malignant cells infiltrating into the subjacent minimal connective tissue stroma with inflammatory cells which led to a provisional diagnosis of verrucous carcinoma of buccal mucosa.

### 2.4. Differential Diagnosis and Its Consideration

Verrucous carcinoma has particular clinical and histopathological features that agree its diagnosis to be obtained, which was an uncharacteristic observation in the present case. First, the clinical reasons for rejection were considered followed by the histological ones for each diagnosis, to obtain a final diagnosis thus clearly separating from others. Oral traumatic fibroma was ruled out since it presents as a firm smooth papule in the mouth, usually the same colour as the rest of the mouth lining, but is sometimes paler or, if it has bled, may look a dark colour. The surface may be ulcerated due to trauma or become rough and scaly. It is usually dome-shaped but may be on a short stalk like a polyp or pedunculated [6]. It should be noted no histological dysplastic features and other observations are not present and fibromas never develop into oral cancer, similarly verrucous hyperplasia (VH) are often associated with papillomas or arise as a de novo lesion. VH (verrucous hyperplasia) and PVL (Proliferative Verrucous Leukoplakia) are irreversible clinicopathologic lesions with considerable potential for evolving into verrucous or squamous cell carcinoma. PVL is a disease of the oral cavity in which VH is a part of its developmental spectrum. Human papillomavirus, as a cofactor, may play an important role in some of these lesions [7, 8]; however, no signs suggestive of HPV infection were observed in the present histopathology section.

### 2.5. Treatment and Follow-Up

**Figure 5:**
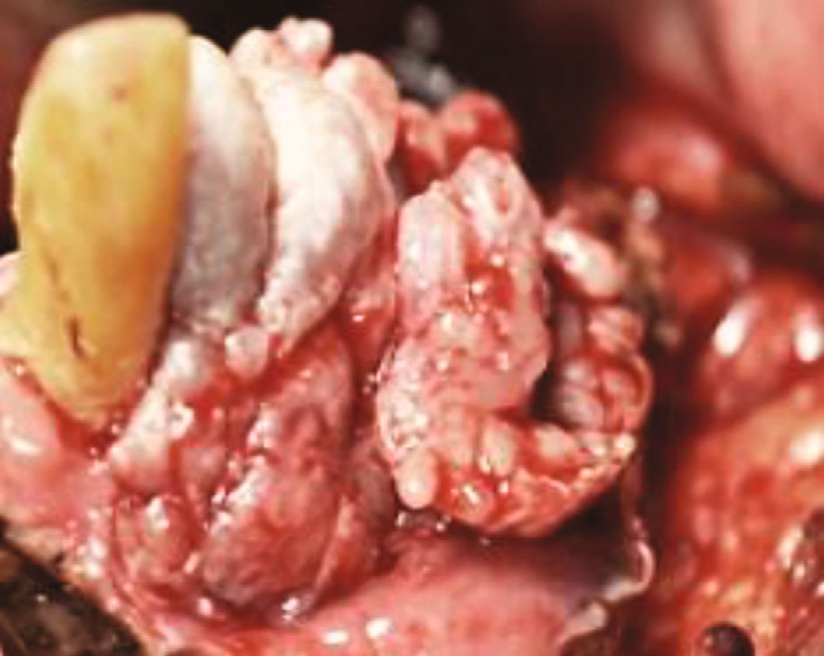
Clinical image showing a wide local surgical excision performed under local anaesthesia.

In the present case, as no other findings like nodal involvement or metastasis were observed, clinical staging of T2N0M0 was given. A wide local surgical excision was planned and performed under local anaesthesia (Figure 5). To ensure complete removal of the lesion with clear margins, further examination of the complete surgical specimen was sent for histopathological investigation and also to rule out infiltrating squamous cell carcinoma. The procedure was uneventful, and postoperative instructions were given. A postoperative follow-up was performed at an interval of every 15 days over a period of 3 months. No further complaints were observed.

### 2.6. Final Diagnosis

**Figure 6:**
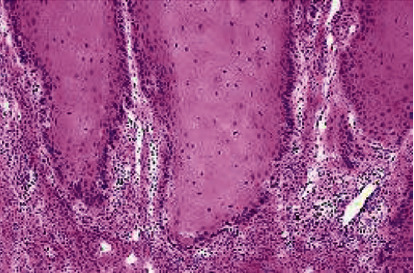
Histopathology of completely excised specimen showed verrucous configuration with a broad bulbous rete pegs with pushing margins in many areas along with features of mild dysplasia and few areas of parakeratin plugging (H&E, original magnification: ×40).

In this instance, though varied clinical course of the disease was noted, the histological result of the complete surgical specimen showed features of verrucous carcinoma similar to the reports obtained during the incisional biopsy thus ruling out squamous cell carcinoma evolved from a verrucous carcinoma (Figure 6). Thus, on complete evaluation of clinical and histological findings, a conclusive diagnosis of verrucous carcinoma (VC) was given.

## 3. Discussion

Verrucous carcinoma (VC) is a rare, low-grade, well-differentiated SCC of the skin or mucosa presenting with a verrucoid or cauliflower-like presence. It shows in the vicinity aggressive behaviour and has low metastatic potential, a low grade of dysplasia, and a good prognosis. It is a tumor that tends to erode more than infiltrate with predominantly horizontal growth and does not present with distant metastasis. It has been presented with various names such as Ackerman tumor and carcinoma cuniculatum which are correlated to specific anatomic sites [9, 10].

In the oral cavity, verrucous carcinoma constitutes 2% to 4.5% of all forms of SCC seen mainly in men older than 50 years and also is associated with a high incidence (37.7%) of a second primary tumor mainly in the oral mucosa (e.g., tongue, lips, palate, and salivary gland) [11]. Kalsotra et al. reported a male to female ratio of 3.6 to 1 in patients with verrucous carcinoma, with a mean age of 53.9 years. In the present report of a case, it is seen with female older than 50 years though it is thought to predominantly affect elderly men [12]. The etiology of VC is related to various local and systemic sources such as Human Papilloma virus and chemicals from cigarettes (smoking) or physical source such as constant trauma and irritation [13, 14]. In this case, the patient did not present with most common factors like tobacco, alcohol consumption, or any occupational factors but presented with history of irritation to the buccal mucosa by tooth.

However, epidemiological studies generally analyse the relationship between chronic traumas of the oral mucosa originated by dentures and cancer, but mostly, they do not include other traumatic factors such as defective teeth or parafunctional habits, and they do not analyse the relationship between dental factors and oral potentially malignant disorders (OPMD). Although faulty restorations, sharp teeth, and ill-fitting dentures have been implicated in a few epidemiological studies, it is not clear whether confounding factors like tobacco and alcohol have been addressed in these studies [15, 16].

Valente et al. [17], Tornes et al. [18], and Rahali et al. [19] reported few cases of squamous cell carcinoma misdiagnosed as a denture-related traumatic ulcer. Meanwhile, de Sant'Ana dos Santos et al. reported misdiagnosis of lip SCC as actinic cheilitis [20]. A case of gingival SCC masquerading as an aphthous ulcer was also reported by Kumari et al. This time elapse might jeopardize patients' overall prognosis; therefore, attempts should be done for timely diagnosis by more logical routes such as decision trees rather than test-and-error methods [6]. The present case scenario admits the fact that other traumatic factors such as sharp teeth or defective teeth are a possible agent for arising of lesion but the primary etiology of trauma as a true etiological factor has to be analysed further by investigation on a larger scale by reporting of various cases across the world.

Histopathologically, VC frequently presents with a hyperplastic epithelium with abundant keratin superficially projecting as exophytic church-spire keratosis with bulbous well-oriented rete ridges shows an endophytic growth pattern with pushing borders. Rapid transition from a normal epithelium to endophytic ingrowth is taken as a significant feature to differentiate it from benign verrucous growths [21]. A classic case of VC shows minimal or no pleomorphism of cells, and no mitotic activity above the basal and suprabasal layers of the epithelium and lymphoplasmacytic inflammatory host reaction are marked, especially in cases where keratin has plunged deep into the connective tissue inducing foreign body granuloma formation as not seen or appreciated in the present case. Insufficient depth of the section, the absence of adjacent normal epithelium, presence of dysplastic features, and evidence of micro invasion create a dilemma for the pathologist. To avoid such scenario, deeper sections were taken and conclusive evidences were considered as it becomes mandatory to rule out SCC [22, 23].

Among various therapeutic approaches, surgical intervention is the primary therapeutic mode to treat verrucous carcinoma. Surgical excision with adequate margins has been proven to be effective in the management of these tumors, and radiotherapy has generally been recommended in the presence of adverse risk factors in the histopathology report such as metastatic lymph node positivity and invasion to the deeper vital structures like nerves and blood vessels [24, 25]. In the present case as no other findings like nodal involvement or metastasis was observed, clinical stating of T2N0M0 was given. Only surgical intervention for complete removal of tumor by intraoral approach was recommended since it may not be effective as in the case of larger area extension of the carcinogenic lesion.

## 4. Conclusion

In the present case, an unexpected or unusual demonstration of verrucous carcinoma caused by local irritation in the form of tooth, with rapid progression of the disease lesser than the usual demonstration even after removal of the etiological agent, suggests a new variation in a disease process. VC should be considered in the differential diagnosis of slow growing ulcerative lesion though not characteristic as noted in the present case considering the evolving epidemiological and etiopathological trends and in addition to the fact that these findings could shed new light on the possible pathogenesis of verrucous carcinoma. In this case, proper diagnosis was made as early as possible to avoid further complication and was appropriately treated. Thus, both clinicians and pathologists must be cautious about chronic traumatic ulcers or ulcerative lesions of the oral cavity and propose diagnostic alternatives to ensure the most appropriate treatment to be given at the right time.

## References

[1] Finkelstein M. W. (2010). A guide to the clinical differential diagnoses of oral mucosal lesions. *CrestOral-Bat dentalcare.com*.

[2] Mortazavi H., Safi Y., Baharvand M., Rahmani S. (2016). Diagnostic features of common oral ulcerative lesions: an updated decision tree. *International journal of dentistry*.

[3] Muñoz‐Corcuera M., Esparza‐Gómez G., González‐Moles M. A., Bascones‐Martínez A. (2009). Oral ulcers: clinical aspects. A tool for dermatologists. Part I. Acute ulcers. *Clinical and Experimental Dermatology*.

[4] Bruce A. J., Dabade T. S., Burkemper N. M. (2015). Diagnosing oral ulcers. *Journal of the American Academy of Physician Assistants*.

[5] Khandekar P. S., Bagdey P. S., Tiwari R. R. (2006). Oral cancer and some epidemiological factors: a hospital based study. *Indian Journal of Community Medicine*.

[6] Kumari P. S., Kumar G. P., Bai Y. D., Reddy E. Y. (2013). Gingival squamous cell carcinoma masquerading as an aphthous ulcer. *Journal of Indian Society of Periodontology*.

[7] Gonsalves W. C., Chi A. A., Neville B. W. (2007). Common oral lesions: part II. Masses and neoplasia. *American family physician*.

[8] Lederman D. A., Fornatora M. L. (2002). *Oral fibromas and fibromatoses*.

[9] Stoopler E. T., Alawi F. (2008). Clinicopathologic challenge: a solitary submucosal mass of the oral cavity. *International Journal of Dermatology*.

[10] Zanini M., Wulkan C., Paschoal F. M., Maciel M. H. M., Machado Filho C. D.´. A. S. (2004). Carcinoma verrucoso: uma variante clínico-histopatológica do carcinoma espinocelular. *Anais Brasileiros de Dermatologia*.

[11] Chaudhary S., Bansal C., Ranga U. (2017). Verrucous carcinoma of the buccal mucosa with extension to the cheek. *Cutis*.

[12] Kalsotra P., Manhas M., Sood R. (2000). Verrucous carcinoma of hard palate. *JK Science*.

[13] Peng Q., Wang Y., Quan H., Li Y., Tang Z. (2016). Oral verrucous carcinoma: from multifactorial etiology to diverse treatment regimens (review). *International journal of oncology*.

[14] Kamath V. V., Varma R. R., Gadewar D. R., Muralidhar M. (1989). Oral verrucous carcinoma: an analysis of 37 cases. *Journal of Cranio-Maxillofacial Surgery*.

[15] Chen B. L., Lin C. C., Chen C. H. (2000). Oral verrucous carcinoma: an analysis of 73 cases. *Clinical Journal of Oral and Maxillofacial Surgery*.

[16] Warnakulasuriya S. (2009). Causes of oral cancer - an appraisal of controversies. *British Dental Journal*.

[17] Valente V. B., Takamiya A. S., Ferreira L. L. (2016). Oral squamous cell carcinoma misdiagnosed as a denture-related traumatic ulcer: a clinical report. *The Journal of Prosthetic Dentistry*.

[18] Tornes K., Bang G., Koppang H. S., Pedersen K. N. (1985). Oral verrucous carcinoma. *International Journal of Oral Surgery*.

[19] Rahali L., Omor Y., Mouden K. (2015). Oral verrucous carcinoma complicating a repetitive injury by the dental prosthesis: a case report. *The Pan African Medical Journal*.

[20] dos Santos F. D., Isper M. A., Novo-Neto J. P., Marqueti A. C., Pereira C. P., Isper F. G. (2012). Misdiagnosis of lip squamous cell carcinoma. *RSBO Revista Sul-Brasileira de Odontologia*.

[21] Piemonte E. D., Lazos P. J., Brunotto M. (2010). Relationship between chronic trauma of the oral mucosa, oral potentially malignant disorders and oral cancer. *Journal of Oral Pathology & Medicine*.

[22] Shergill A. K., Solomon M. C., Carnelio S., Kamath A. T., Aramanadka C., Shergill G. S. (2015). Verrucous carcinoma of the oral cavity: current concepts. *International Journal of Scientific Study*.

[23] Varshney S., Singh J., Saxena R. K., Kaushal A., Pathak V. P. (2004). Verrucous carcinoma of larynx. *Indian Journal of Otolaryngology and Head and Neck Surgery*.

[24] McClure D. L., Gullane P. J., Slinger R. P., Wysocki G. P. (1984). Verrucous carcinoma-changing concepts in management. *The Journal of Otolaryngology*.

[25] Asha M. L., Vini K., Chatterjee I., Patil P. (2014). Verrucous carcinoma of buccal mucosa: a case report. *International Journal of Health Sciences*.

